# The Impact of MOUD Initiation in Patients With Injection Drug Use-Associated Infective Endocarditis

**DOI:** 10.1093/ofid/ofag288

**Published:** 2026-05-16

**Authors:** Seth Losiewicz, Bennett Collis, Sami El-Dalati, Evan Hall, Chloe Cao, Meredith Johnson, Takaaki Kobayashi, Bobbi Jo Stoner, Daniel Weaver

**Affiliations:** Department of Internal Medicine, University of Kentucky Medical Center, Lexington, Kentucky, USA; University of Kentucky College of Medicine, Lexington, Kentucky, USA; Division of Infectious Diseases, Department of Internal Medicine, University of Kentucky Medical Center, Lexington, Kentucky, USA; Department of Internal Medicine, University of Kentucky Medical Center, Lexington, Kentucky, USA; Department of Internal Medicine, HCA Healthcare/USF Morsani College of Medicine GME/HCA Florida Trinity, Tampa, Florida, USA; Department of Surgery, University of Kentucky Medical Center, Lexington, Kentucky, USA; Division of Infectious Diseases, Department of Internal Medicine, University of Kentucky Medical Center, Lexington, Kentucky, USA; University of Kentucky College of Pharmacy, Lexington, Kentucky, USA; Division of Hospital Medicine, Department of Internal Medicine, University of Kentucky Medical Center, Lexington, Kentucky, USA

**Keywords:** endocarditis, injection drug use, medications for opioid use disorder, multidisciplinary teams

## Abstract

This single-center retrospective study evaluated 218 patients with injection drug use-associated infective endocarditis, 169 of whom received medications for opioid use disorder (MOUD). Initiation of MOUD was associated with a nonsignificant increase in completion of antibiotic treatment and a significant reduction in 30-day postdischarge mortality, but not 90-day mortality.

Hospitalizations for infections related to injection drug use (IDU) have more than doubled in the past 2 decades [1, 2]. Correspondingly, the incidence of infective endocarditis (IE) in patients who inject drugs (PWIDs) has increased significantly since 2003 and is associated with sepsis, the need for surgical valve replacements, and death [3]. In 2018 alone, there were an estimated 3 694 500 PWIDs most of whom have a concurrent opioid use disorder (OUD) [4, 5]. Medications for opioid use disorder (MOUD), which include buprenorphine products and methadone, have been shown to substantially reduce the risk of overdose and mortality rates in patients with OUD. There is less data regarding the impact of MOUD on mortality in patients with IDU-related infective endocarditis (IDU-IE), although there may be a decrease in short-term mortality with MOUD initiation [2]. Multidisciplinary endocarditis teams (MDETs) have also been associated with improved short-term outcomes in patients with IE [6]. Given the potential mortality benefit of MOUD and the increasing incidence of IDU-IE, MOUD may represent an important therapeutic option, yet remains underutilized in this population [1] Furthermore, there is little data on MOUD initiation in the setting of an MDET. We report our approximately 4-year experience using an MDET to manage IDU-IE and assess rates of MOUD initiation and their impact on mortality and retention in care compared with patients who did not receive MOUD.

## METHODS

Institutional review board (IRB) approval was obtained from the University of Kentucky. Patient consent was not required by the IRB. In September 2021, University of Kentucky Healthcare (UK) created a multidisciplinary endocarditis team (MDET) and cardiovascular infectious diseases consult service (CVIDCS). The structure and function of the MDET and CVIDCS have been explained in detail previously [7, 8]. All patients admitted to UK with IE are seen by the CVIDCS and discussed at the weekly MDET conference. All patients with active substance use disorder or a history of IDU are offered addiction medicine consultation. The addiction medicine team meets with the patient to obtain history about their previous substance use, past experience with MOUD, including whether any particular agent was or was not successful, and what factors contributed to this. The addiction team will offer patients the options of micro-induction or standard induction with buprenorphine, methadone, or naltrexone. Patients who elect to initiate buprenorphine are also offered the option to transition to a long-acting injectable form of buprenorphine at the time of discharge and the first dose of this medication is provided on the day the patient leaves the hospital. If patients elect to continue on oral or sublingual buprenorphine they are discharged with the combination product buprenorphine/naloxone and follow-up is scheduled with either the UK addiction clinic or a provider closer to the patient's home. “Bridge” prescriptions of up to 7 days are provided until patients can establish with their new clinic. For patients interested in methadone, the team explores whether the patient can reliably transport daily to a clinic near their home prior to starting therapy. The addiction consult team schedules all outpatient methadone intake appointments prior to discharge. The addiction medicine team also attends all MDET conferences and discusses the patients’ preferred treatment strategy as well as any social or logistical barriers that may impact the hospital stay and/or longitudinal follow-up.

Beginning in September 2021, a registry was created in the hospital's electronic medical record containing all patients presented at the weekly MDET conference. Investigators retrospectively collected patients’ demographic, comorbidities, diagnostics, treatments, and outcomes data. For patients who did not follow-up in clinic, data for mortality were collected based on emergency department visits and admissions to either the authors’ institution or other hospitals in the region using shared electronic medical records. Follow-up data at 90 days was available for all patients. Recent IDU was defined as any injection substance use within 30 days of the index hospitalization as self-reported by patients on chart review of hospital notes. Previous endocarditis admissions were defined as any prior admission for endocarditis at any point. Patients who died during the index hospitalization were excluded from this study.

Patients were included if they met modified Duke criteria for definite IE and Diagnostic and Statistical Manual of Mental Disorders-5 criteria for OUD [9, 10]. Primary outcomes were 30- and 90-day mortality and readmission rates. Secondary outcomes included completion of antibiotic therapy as reported by patients, and follow-up rates.

Three separate analyses were conducted: 1 evaluating antibiotic completion, 1 evaluating 30-day all-cause mortality after hospital discharge, and 1 evaluating 90-day all-cause mortality. For antibiotic completion, univariable logistic regression models were first fitted for each candidate variable. Variables with a *P* value < .05 in univariable analyses were included in the multivariable model. For the mortality analyses, univariable logistic regression models were first fitted for each candidate variable, with 30-day or 90-day mortality as the dependent variable. Variables with a *P* value < .05 in univariable analyses were included in the multivariable model. As receipt of MOUD was the primary variable of interest, it was included in all multivariable models regardless of its *P* value in univariable analyses. Multivariable logistic regression was performed using Firth's penalized likelihood method to reduce small-sample bias and account for potential data separation. In the logistic regression analyses, central nervous system complications were counted once per patient and included mycotic aneurysm, stroke, and intracranial hemorrhage. Results are reported as odds ratios (ORs) with 95% confidence intervals (CIs) and 2-sided *P* values; statistical significance was defined as *P* < .05. All analyses were performed using SAS version 9.4 (SAS Institute Inc., Cary, NC).

## RESULTS

### Patient Characteristics

Between 7 September 2021 and 1 March 2025, a total of 226 patients with IE and opioid use disorder (OUD) were identified. Eight patients died during hospitalization and were excluded, leaving 218 patients included in the analytic sample; 169 patients were initiated on MOUD during the index hospitalization and 49 were not (Supplementary Figure 1). The 2 groups were generally similar with respect to age, sex, race, and several baseline clinical characteristics (Table 1). A history of IDU was observed in 82.2% of patients in the MOUD group and 69.4% in the non-MOUD group (*P* = .05). Diabetes mellitus was present in 5.3% of the MOUD group and 14.3% of the non-MOUD group (*P* = .03), while intracranial hemorrhage occurred in 8.9% and 22.4%, respectively (*P* = .01). Regarding valvular involvement, aortic valve vegetations were identified in 21.3% of the MOUD group and 34.7% of the non-MOUD group (*P* = .05), and multivalvular involvement was present in 10.1% and 22.4%, respectively (*P* = .02). Involvement of the mitral, tricuspid, and pulmonic valves did not differ significantly between groups. Rates of prior endocarditis, hepatitis C viremia, HIV infection, prosthetic valve presence, Pittsburgh bacteremia score, ischemic stroke, and valve surgery were similar between groups. Among patients who received MOUD, 126 (74.6%) were treated with buprenorphine/naloxone, 37 (21.9%) with methadone, 4 (3.2%) with buprenorphine monotherapy, and 1 (0.6%) with oral naltrexone. At discharge, 36 patients (21.3%) received long-acting injectable buprenorphine and 1 patient (0.6%) received injectable naltrexone.

**Table 1.** Demographic and Infection Related Data for Patients With Injection Drug Use Related Endocarditis and Opioid Use Disorder

Variable	MOUD N = 169	No MOUD N = 49	*P* Value
Age, Y, Median (IQR)	36 (31–43)	40 (33–47)	.11
Male, % (n)	53.8 (91)	65.3 (32)	.15
Female, % (n)	46.2 (78)	34.7 (17)	
White, % (n)	96.4 (163)	95.9 (47)	.89
Black, % (n)	3.0 (5)	4.1 (2)	
Other, % (n)	0.6 (1)	0 (0)	
Injection Drug Use, Past 30-days, % (n)	82.2 (139)	69.4 (34)	.05
Previous Endocarditis Admission, % (n)	45.6 (77)	46.9 (23)	.87
Hepatitis C Viremia, % (n)	43.8 (74)	49.0 (24)	.52
HIV infection, % (n)	1.2 (2)	2.0 (1)	.67
Diabetes mellitus, % (n)	5.3 (9)	14.3 (7)	.03
Chronic dialysis, % (n)	1.8 (3)	0 (0)	.35
Prosthetic valve, % (n)	11.8 (20)	18.4 (9)	.23
Addiction medicine consult, % (n)	100 (169)	100 (49)	N/A
Infectious Disease consult, % (n)	100 (169)	100 (49)	N/A
Aortic valve vegetation, % (n)	21.3 (36)	34.7 (17)	.05
Mitral valve vegetation, % (n)	23.1 (39)	26.5 (13)	.62
Tricuspid Valve vegetation, % (n)	55.6 (94)	51.0 (25)	.57
Pulmonic valve vegetation, % (n)	1.2 (2)	2.0 (1)	.67
Multivalvular vegetations, % (n)	10.1 (17)	22.4 (11)	.02
Pittsburgh bacteremia score, median (IQR)	0 (0–2)	0 (0–2)	.42
Ischemic stroke, % (n)	14.2 (24)	18.4 (9)	.47
Intracranial hemorrhage, % (n)	8.9 (15)	22.4 (11)	.01
Valve surgery, % (n)	23.1 (39)	34.7 (17)	.10
Percutaneous mechanical aspiration, % (n)	4.7 (8)	2.0 (1)	.40
Buprenorphine/naloxone induction, % (n)	74.6 (126)	N/A	N/A
Buprenorphine induction % (n)	3.2 (4)	N/A	N/A
Oral methadone, % (n)	21.9 (37)	N/A	N/A
Oral naltrexone, % (n)	0.6 (1)	N/A	N/A
Receipt of long-acting injectable buprenorphine on discharge, % (n)	21.3 (36)	N/A	N/A
Receipt of long-acting injectable naltrexone at discharge, % (n)	0.6 (1)	N/A	N/A

Abbreviations: HIV, human immunodeficiency virus; IQR, interquartile range; MOUD, medications for opioid use disorder; N/A, not applicable.

### Clinical Outcomes

Bivariable clinical outcomes are summarized in Table 2. Patient-directed discharge occurred less frequently in the MOUD group compared with the non-MOUD group (13.6% vs 32.7%; *P* = .002). Completion of the prescribed antibiotic course was higher among patients who received MOUD (81.1% vs 63.3%; *P* = .009). Thirty-day all-cause mortality was lower in the MOUD group compared with the non-MOUD group (1.2% vs 8.2%; *P* = .009), as was 90-day all-cause mortality (4.1% vs 14.3%; *P* = .01). Thirty-day and 90-day all-cause readmission rates were numerically lower in the MOUD group; however, these differences were not statistically significant (30-day: 23.1% vs 30.6%, *P* = .29; 90-day: 30.2% vs 42.9%, *P* = .10). Addiction medicine follow-up was more common among patients in the MOUD group (13.0% vs 2.0%; *P* = .03). Follow-up with infectious diseases, cardiac surgery, and cardiology did not differ significantly between groups. Absence of outpatient follow-up was numerically higher in the non-MOUD group, though this difference did not reach statistical significance (38.8% vs 27.2%; *P* = .12).

**Table 2.** Outcomes Data for Patients With Injection Drug Use Related Endocarditis and Opioid Use Disorder

Variable	MOUD N = 169	No MOUD N = 49	*P* Value
Patient-directed discharge, % (n)	13.6 (23)	32.7 (16)	.002
Length of stay, d, median (IQR)	26 (15–42)	20 (8–37)	.02
Antibiotic completion, % (n)	81.1 (137)	63.3 (31)	.009
30-D mortality, % (n)	1.2 (2)	8.2 (4)	.009
30-D relapsed infection, % (n)	0 (0)	0 (0)	N/A
30-D all-cause Readmission, % (n)	23.1 (39)	30.6 (15)	.29
90-D mortality, % (n)	4.1 (7)	14.3 (7)	.01
90-D relapsed infection, % (n)	0.6 (1)	0 (0)	.59
90-D all-cause readmission, % (n)	30.2 (51)	42.9 (21)	.10
Addiction medicine follow-up, % (n)	13.0 (22)	2.0 (1)	.03
Infectious diseases follow-up, % (n)	63.3 (107)	55.1 (27)	.30
Cardiac surgery follow-up, % (n)	45.6 (77)	49.0 (24)	.68
Cardiology follow-up, % (n)	27.8 (47)	22.4 (11)	.45
No outpatient follow-up, % (n)	27.2 (46)	38.8 (19)	.12

### Antibiotic Completion in Logistic Regression Models

In multivariable logistic regression analyses evaluating antibiotic completion, receipt of MOUD was associated with higher odds of completing the prescribed antibiotic course, although this association did not reach statistical significance (adjusted odds ratio [aOR] 2.04, 95% CI .97–4.26; *P* = .060; Supplementary Table 1). Longer hospital length of stay was independently associated with antibiotic completion (aOR per day 1.05, 95% CI 1.03–1.08; *P* < .001). No other demographic, clinical, or microbiologic variables were independently associated with antibiotic completion in the adjusted model.

### Thirty-day Mortality in Logistic Regression Models

In the multivariable model assessing 30-day all-cause mortality after hospital discharge, receipt of MOUD was independently associated with substantially lower odds of death within 30 days (aOR 0.11, 95% CI .01–.87; *P* = .037; Supplementary Table 2). Completion of the antibiotic course was also strongly protective (aOR 0.04, 95% CI .003–.41; *P* = .008). In contrast, acute renal replacement therapy and prosthetic valve involvement were associated with significantly increased odds of 30-day mortality. Other markers of severity of illness, including central nervous system complications and mechanical ventilation, were not independently associated with mortality in the adjusted analysis.

### Ninety-day Mortality in Logistic Regression Models

At 90 days after hospital discharge, receipt of MOUD was no longer significantly associated with mortality (aOR 0.61, 95% CI .18–2.10; *P* = .43; Supplementary Table 3). Older age remained independently associated with increased 90-day mortality (aOR per year 1.07, 95% CI 1.00–1.13; *P* = .046), as did prosthetic valve involvement (aOR 8.63, 95% CI 1.77–42.16; *P* = .008) and central nervous system complications (aOR 3.80, 95% CI 1.03–14.00; *P* = .045). Completion of the antibiotic course remained protective at 90 days (aOR 0.14, 95% CI .04–.51; *P* = .003).

## DISCUSSION

In this single-center retrospective cohort study of 218 patients with OUD and definite IDU-IE, excluding 8 patients who died during the index hospitalization, initiation of MOUD was associated with a significant reduction in 30-day postdischarge mortality but not with 90-day mortality after multivariable adjustment. Completion of antibiotic therapy was associated with lower mortality at both 30 and 90-days, and initiation of MOUD was associated with higher rates of antibiotic completion, although this association did not reach statistical significance in adjusted analyses. Consistent with prior literature, older age, prosthetic valve involvement, central nervous system complications, and acute renal failure requiring dialysis were independently associated with increased mortality. This study is unique in that it assesses MOUD initiation in IE within the framework of a MDET.

These findings are similar to other studies which found that MOUD initiation was associated with improved short-term mortality but the benefits waned over time [2, 11, 12]. This attenuation may reflect declining treatment adherence or continuity of care after discharge, though other clinical and behavioral factors could contribute. Notably, among patients who started on MOUD, only 13.0% had follow-up with addiction medicine at our institution, while the follow-up rates were 63.3%, 45.6%, and 27.8% for infectious disease, cardiac surgery, and cardiology, respectively. At the authors’ program, many patients with OUD are referred for follow-up in the community for logistical reasons as patients often live in rural areas without the transportation required to return to a tertiary care center. This necessitates transitions to new providers who may have more restrictive MOUD prescribing practices, thereby creating new barriers to care. Given the long-term impact on mortality of OUD and IE, this population may benefit from closer follow-up with more specialized addiction providers. Integrating MOUD treatment across disciplines, particularly infectious disease, may also help improve retention with MOUD as follow-up rates for this specialty were >60%.

On bivariable analysis, patients in the MOUD group also had lower rates of patient-directed discharge, which may have contributed to their ability to complete antibiotic treatment. IDU-IE is associated with high rates of patient-directed discharge and the resulting disruptions in care may contribute to increased mortality by preventing completion of antibiotics or indicated procedures [13–15]. Interventions that decrease the risk of patient-directed discharge may offer the potential to improve patient outcomes. Finally, while the 30-day and 90-day readmission rates were lower in the MOUD group on bivariable analysis, this difference was not statistically significant and other medical and social factors may play a larger role in readmissions.

### Limitations

Our study is limited by its single-center, retrospective nature. Patients were not randomized and there may have been significant selection bias as patients who initiated MOUD may have been more motivated to avoid further IDU. After excluding the 8 patients who died during hospitalization, there were only 6 deaths for 30-day mortality and 14 deaths for 90-day mortality. The small number of outcome events may have affected the stability of the estimates in the logistic regression models.

## CONCLUSION

Initiation of MOUD among patients with IDU-IE was associated with lower 30-day postdischarge mortality, but not with 90-day mortality. MOUD initiation was associated with higher rates of antibiotic completion, although this association did not reach statistical significance. Further research is needed to better understand the factors contributing to this time-limited benefit and to identify care models that improve long-term retention in treatment and outcomes.

## Supplementary Material

ofag288_Supplementary_Data

## Supplementary Data

Supplementary materials are available at *Open Forum Infectious Diseases* online. Consisting of data provided by the authors to benefit the reader, the posted materials are not copyedited and are the sole responsibility of the authors, so questions or comments should be addressed to the corresponding author.
