# Supplementary material for: The Impact of MOUD Initiation in Patients With Injection Drug Use-Associated Infective Endocarditis
Source: Open Forum Infect Dis. 2026 May 16;13(5):ofag288. doi: 10.1093/ofid/ofag288 (PMC13197196; doi:10.1093/ofid/ofag288)
Supplement: ofag288_Supplementary_Data [file ofag288_supplementary_data.zip › MOUD Supplemental Appendix Revised v2.docx]

Supplemental Table1. Univariable and multivariable logistic regression models identifying factors associated with antibiotics completion among patients with infective endocarditis (n=218).

Abbreviations: OR, odds ratio; CI, confidence interval; HIV, human immunodeficiency virus; ICU, intensive care unit; NA, not applicable.

|  | **Univariable** | | **Multivariable** | |
| --- | --- | --- | --- | --- |
| **Variable** | **OR (95% Cl)** | **P-value** | **OR (95% Cl)** | **P-value** |
| Age | 1.015 (0.980–1.051) | 0.4045 |  |  |
| Medications for opioid use disorder | 2.486 (1.239–4.990) | 0.0104 | 2.035 (0.972–4.261) | 0.0595 |
| Acute renal replacement therapy | 0.570 (0.185–1.752) | 0.3262 |  |  |
| AngioVac procedure performed | 1.043 (0.210–5.187) | 0.9590 |  |  |
| Chronic dialysis | 0.590 (0.052–6.642) | 0.6692 |  |  |
| Dental source identified | 0.786 (0.416–1.483) | 0.4567 |  |  |
| Diabetes mellitus | 0.464 (0.160–1.348) | 0.1581 |  |  |
| Mood disorder | 1.022 (0.489–2.139) | 0.9534 |  |  |
| Pitt bacteremia score | 1.087 (0.906–1.305) | 0.3697 |  |  |
| Length of stay (days) | 1.056 (1.030–1.083) | <0.0001 | 1.051 (1.025–1.077) | **<0.0001** |
| HIV infection | 0.590 (0.052–6.642) | 0.6692 |  |  |
| Cardiac implantable electronic device | NA | NA |  |  |
| Male compared to female | 0.643 (0.341–1.213) | 0.1729 |  |  |
| Cardiac device extraction performed | NA | NA |  |  |
| Hepatitis C virus | 0.947 (0.502–1.784) | 0.8655 |  |  |
| Heart failure at presentation | 0.993 (0.527–1.872) | 0.9834 |  |  |
| Intensive care unit admission | 1.860 (0.978–3.536) | 0.0583 |  |  |
| Vasopressor requirement | 1.267 (0.610–2.629) | 0.5258 |  |  |
| Injection drug use | 0.834 (0.370–1.879) | 0.6616 |  |  |
| Valvular vegetation present | 0.887 (0.281–2.804) | 0.8382 |  |  |
| Multivalvular Vegetations | 1.917 (0.632–5.812) | 0.2504 |  |  |
| Central nervous system complication | 1.275 (0.652–2.495) | 0.4781 |  |  |
| Mechanical ventilation required | 1.755 (0.901–3.422) | 0.0984 |  |  |
| Previous infective endocarditis | 0.895 (0.475–1.685) | 0.7309 |  |  |
| Prosthetic valve involvement | 1.499 (0.541–4.158) | 0.4363 |  |  |
| Non-White race compared to White Race | NA | NA |  |  |
| Septic pulmonary emboli | 0.907 (0.480–1.711) | 0.7623 |  |  |
| Cardiac surgery performed | 2.100 (0.919–4.801) | 0.0786 |  |  |

Supplemental Table 2. Univariable and multivariable logistic regression models identifying factors associated with 30-day mortality among patients with infective endocarditis. (n=218)

Abbreviations: OR, odds ratio; CI, confidence interval; HIV, human immunodeficiency virus; ICU, intensive care unit; NA, not applicable.

|  | **Univariable** | | **Multivariable** | |
| --- | --- | --- | --- | --- |
| **Variable** | **OR (95% Cl)** | **P-value** | **OR (95% Cl)** | **P-value** |
| Age | 1.046 (0.969 – 1.129) | 0.2472 |  |  |
| Medications for opioid use disorder | 0.135 (0.024 – 0.759) | 0.0231 | 0.109 (0.014 – 0.874) | **0.0369** |
| Acute renal replacement therapy | 16.667 (3.036 – 91.511) | 0.0012 | 22.477 (2.538 – 199.087) | **0.0052** |
| Antibiotic course completed | 0.054 (0.006 – 0.473) | 0.0084 | 0.035 (0.003 – 0.412) | **0.0077** |
| AngioVac procedure performed | NA | NA |  |  |
| Chronic dialysis | NA | NA |  |  |
| Dental source identified | 1.926 (0.345 – 10.740) | 0.4548 |  |  |
| Diabetes mellitus | 2.627 (0.288 – 23.951) | 0.3918 |  |  |
| Mood disorder | 0.615 (0.070 – 5.388) | 0.6610 |  |  |
| Pitt bacteremia score | 1.230 (0.900 – 1.681) | 0.1937 |  |  |
| Length of stay (days) | 0.962 (0.905 – 1.022) | 0.2115 |  |  |
| HIV infection | NA | NA |  |  |
| Cardiac implantable electronic device | NA | NA |  |  |
| Male compared to Female | 1.563 (0.280 – 8.719) | 0.6106 |  |  |
| Cardiac device extraction performed | NA | NA |  |  |
| Hepatitis C virus | 0.604 (0.108 – 3.370) | 0.5656 |  |  |
| Heart failure at presentation | NA | NA |  |  |
| Intensive care unit admission | NA | NA |  |  |
| Vasopressor requirement | 5.571 (0.993 – 31.256) | 0.0509 |  |  |
| Injection drug use | 0.494 (0.088 – 2.789) | 0.4246 |  |  |
| Valvular vegetation present | NA | NA |  |  |
| Multivalvular Vegetations | 1.370 (0.154 – 12.179) | 0.7774 |  |  |
| Central nervous system complication | 3.654 (0.654 – 20.414) | 0.1399 |  |  |
| Mechanical ventilation required | 7.184 (0.825 – 62.565) | 0.0742 |  |  |
| Previous infective endocarditis | 6.158 (0.707 – 53.610) | 0.0997 |  |  |
| Prosthetic valve involvement | 7.154 (1.371 – 37.328) | 0.0196 | 17.004 (1.871 – 154.498) | **0.0118** |
| Non-White race compared to White Race | NA | NA |  |  |
| Septic pulmonary emboli | 1.719 (0.308 – 9.589) | 0.5366 |  |  |
| Cardiac surgery performed | 0.571 (0.065 – 4.995) | 0.6125 |  |  |

Supplemental Table 3. Univariable and multivariable logistic regression models identifying factors associated with 90-day mortality among patients with infective endocarditis(n=218).

Abbreviations: OR, odds ratio; CI, confidence interval; HIV, human immunodeficiency virus; ICU, intensive care unit; NA, not applicable.

|  | **Univariable** | | **Multivariable** | |
| --- | --- | --- | --- | --- |
| **Variable** | **OR (95% Cl)** | **P-value** | **OR (95% Cl)** | **P-value** |
| Age | 1.072 (1.018 – 1.128) | 0.0089 | 1.065 (1.001 – 1.132) | **0.0459** |
| Medications for opioid use disorder | 0.259 (0.086 – 0.780) | 0.0163 | 0.612 (0.179 – 2.095) | 0.4343 |
| Acute renal replacement therapy | 4.364 (1.072 – 17.758) | 0.0396 | 4.199 (0.676 – 26.073) | 0.1236 |
| Antibiotic course completed | 0.194 (0.064 – 0.591) | 0.0039 | 0.144 (0.041 – 0.507) | **0.0025** |
| AngioVac procedure performed | NA | NA |  |  |
| Chronic dialysis | NA | NA |  |  |
| Dental source identified | 2.500 (0.759 – 8.230) | 0.1318 |  |  |
| Diabetes mellitus | 2.262 (0.460 – 11.119) | 0.3149 |  |  |
| Mood disorder | 0.840 (0.225 – 3.132) | 0.7951 |  |  |
| Pitt bacteremia score | 1.108 (0.866 – 1.418) | 0.4143 |  |  |
| Length of stay | 0.976 (0.942 – 1.011) | 0.1807 |  |  |
| HIV infection | NA | NA |  |  |
| Cardiac implantable electronic device | NA | NA |  |  |
| Male compared to Female | 2.013 (0.611 – 6.631) | 0.2499 |  |  |
| Cardiac device extraction performed | NA | NA |  |  |
| Hepatitis C virus | 1.242 (0.420 – 3.669) | 0.6953 |  |  |
| Heart failure at presentation | 3.166 (0.961 – 10.429) | 0.0581 |  |  |
| Intensive care unit admission | 1.731 (0.561 – 5.343) | 0.3401 |  |  |
| Vasopressor requirement | 2.849 (0.955 – 8.503) | 0.0605 |  |  |
| Injection drug use | 0.922 (0.246 – 3.458) | 0.9045 |  |  |
| Valvular vegetation present | NA | NA |  |  |
| Multivalvular Vegetations | 1.953 (0.510 – 7.483) | 0.3289 |  |  |
| Central nervous system complication | 4.891 (1.480 – 16.163) | 0.0092 | 3.800 (1.031 – 14.002) | **0.0449** |
| Mechanical ventilation required | 2.624 (0.849 – 8.110) | 0.0938 |  |  |
| Previous infective endocarditis | 1.623 (0.544 – 4.846) | 0.3854 |  |  |
| Prosthetic valve involvement | 4.167 (1.289 – 13.469) | 0.0171 | 8.634 (1.770 – 42.160) | **0.0077** |
| Non-White race compared to White race | NA | NA |  |  |
| Septic pulmonary emboli | 1.569 (0.508 – 4.843) | 0.4337 |  |  |
| Cardiac surgery performed | 1.169 (0.352 – 3.888) | 0.7987 |  |  |

Supplemental Figure 1 Alt Text:

730 patients were managed by a multidisciplinary endocarditis team and 512 patients were excluded, including those with possible or rejected endocarditis, patients without opioid use disorder and those who died during the index hospitalization. Of the remaining 218 patients with definite endocarditis and opioid use disorder, 169 patients were initiated on medications for opioid use disorder and 49 patients were not.
