# Supplementary figures and images for: The Impact of MOUD Initiation in Patients With Injection Drug Use-Associated Infective Endocarditis
Source: Open Forum Infect Dis. 2026 May 16;13(5):ofag288. doi: 10.1093/ofid/ofag288 (PMC13197196; doi:10.1093/ofid/ofag288)

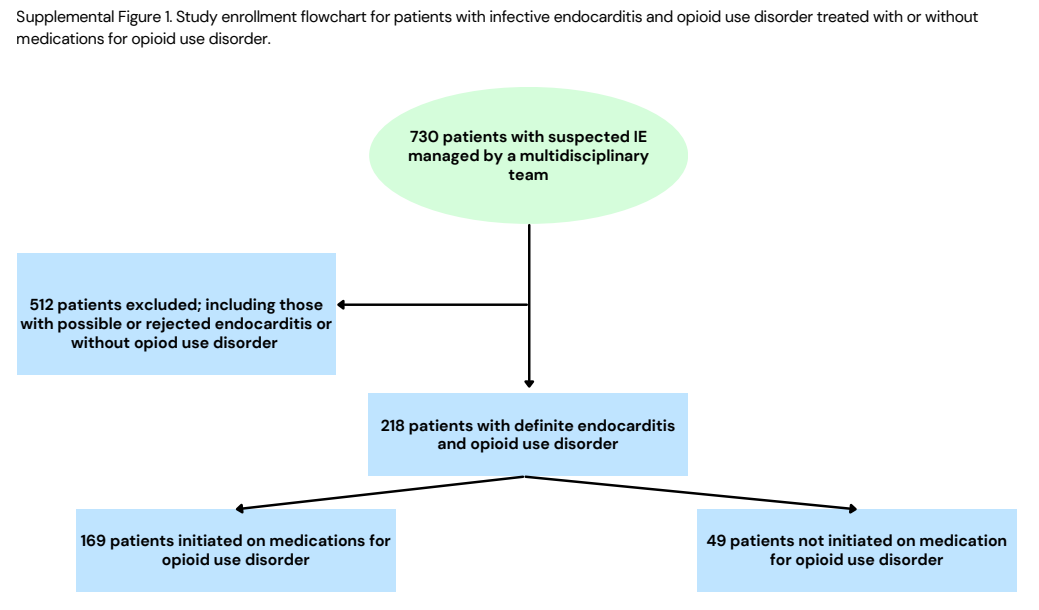

Supplement: ofag288_Supplementary_Data [file ofag288_supplementary_data.zip › MOUD Supplemental Figure 1 (2).tiff]
